# Editorial: Celiac disease in children

**DOI:** 10.3389/fped.2025.1703676

**Published:** 2025-10-09

**Authors:** Yasin Sahin, Nafiye Urganci, Michal Kori, Eylem Sevinc

**Affiliations:** ^1^ Clinic of Pediatric Gastroenterology, Gaziantep City Hospital, Gaziantep, Türkiye; ^2^ Department of Pediatric Gastroenterology, Faculty of Medicine, Gaziantep Islam Science and Technology University, Gaziantep, Türkiye; ^3^ Department of Pediatric Gastroenterology, SBU Sisli Etfal Training and Research Hospital, Istanbul, Türkiye; ^4^ Department of Pediatric Gastroenterology, Kaplan Medical Center, Rehovot, Israel; ^5^ Faculty of Medicine, Hebrew University of Jerusalem, Israel; ^6^ Department of Pediatric Gastroenterology, Faculty of Medicine, Karabuk University, Karabuk, Türkiye

**Keywords:** celiac disease, children, diagnosis, prevalence, screening

**Editorial on the Research Topic**
Celiac disease in children

The aim of this Research Topic was to gather original research articles, case reports, and review articles focusing on various aspects of pediatric celiac disease (CD) in 2025 and contribute to the existing literature. This editorial reviewed 17 articles, 11 of which were accepted for publication by the reviewers and Editors in the Research Topic “*Celiac Disease In Children*”. The articles cover topics such as the prevalence of acute reactions to inadvertent gluten contamination, molar incisor hypomineralization, serum levels of vitamin D and calcium-phosphorus, allergic and immunological evaluations, clinical manifestations, a non-biopsy strategy in children with CD, celiac disease screening and depression, and anxiety in adolescents with CD.

Celiac disease is an immune-mediated enteropathy triggered by dietary gluten consumption in genetically predisposed individuals. It is marked by the presence of specific antibodies and variable degrees of small intestinal mucosal damage (1). In addition to genetic susceptibility and gluten exposure, the pathogenesis of CD autoimmunity is considered to be multifactorial, including impaired intestinal barrier function, a gluten-induced proinflammatory innate immune response, and an inappropriate adaptive immune response (1, 2). Intestinal fatty acid binding protein (i-FABP) and fecal zonulin (FZ) are crucial for preserving intestinal physiological functions and may indicate enterocyte damage (Geller et al.). A relationship has been shown between the clinical manifestations of CD and the levels of FZ and i-FABP (Geller et al.). The authors stated that increased FZ and i-FABP values can serve as markers of increased intestinal barrier permeability and damage, opening up new possibilities for understanding the restoration processes of the small intestinal mucosa.

Due to increased physician awareness and the extensive use of highly sensitive and specific diagnostic tests for CD, its prevalence rate has dramatically increased over the past three decades (1). Approximately 95% of cases of CD remain undiagnosed despite increased awareness and the availability of trustworthy testing techniques (1, Naredi Scherman et al.).

The clinical presentation of CD varies considerably, and there has been a notable change in how CD presents itself over the last three decades. Infants typically exhibit different symptoms than older children. Infants may present with malabsorptive symptoms, including diarrhea, anorexia, abdominal distension, and failure to thrive. Young children may present with any of the above symptoms and/ or abdominal pain and iron deficiency anemia, which are the most common presenting symptoms in this age group. While the symptoms of older children are either limited or atypical, some develop non-specific gastrointestinal symptoms, such as constipation, along with extraintestinal symptoms and signs such as short stature, iron deficiency, and delayed puberty. In older children and adults common presentations include infertility, dermatitis herpetiformis, osteoporosis, enamel defects, and neurological manifestations such as ataxia, anxiety, and recurrent headaches (1, 3). Many children are diagnosed without exhibiting symptoms due to the screening of family members with CD, or the screening of patients with associated autoimmune or genetic diseases.

Consistent with the literature, the most prevalent symptoms among children with CD include abdominal pain, diarrhea, and failure to thrive, all of which were found to be common in a recent study conducted in Lebanon (1, 4, Andari et al.). In addition to having considerably lower vitamin D levels and higher tissue transglutaminase levels, children recently identified as having CD and who were found to be incompatible with a gluten-free diet were also observed to be more likely to have molar-incisor hypomineralization (Tok et al.). Approximately 75% of patients with CD are found to have osteopenia, and up to 30% exhibit osteoporosis, as a result of inadequate absorption of calcium and vitamin D consequent to the mucosal damage (1, 5).

Adequate exposure to sunlight is known to increase vitamin D levels. However, Kamilova et al. found that a high prevalence of vitamin D deficiency is also seen in children with CD living in regions with increased sunlight exposure.

Individuals with autoimmune thyroid disorders, selective immunoglobulin A (IgA) deficiency, type 1 diabetes mellitus, or psoriasis, along with those with genetic syndromes such as Turner syndrome, Down syndrome, or Williams syndrome, are recognized as being at increased risk of developing the disease (1, Lattuada et al.). Patients with these conditions should always be evaluated for CD because of these known correlations.

Children with CD may potentially have immunologic abnormalities and allergic disorders. Beyond the well-known correlation with selective IgA deficiency, a recent study demonstrated a notable prevalence of immunologic abnormalities (e.g., partial IgM deficiency, unclassified hypogammaglobulinemia, etc.) and allergic diseases (e.g., aeroallergen sensitivity, allergic rhinitis, allergic conjunctivitis, food allergy, and asthma) in children with CD (Demirtaş Güner and Baskın).

The 2020 ESPGHAN guidelines recommend that CD be diagnosed without an intestinal biopsy under certain conditions (6). According to a recent study from Romania, serology-based diagnosis results in less compliance with follow-up, more dietary violations, and shorter mucosal healing than in patients with biopsy-proven CD (Enache et al.). Additionally, the authors suggested that management should be improved, paying particular attention to individuals who were diagnosed with the non-biopsy method according to the new ESPGHAN guidelines (Enache et al.).

A wide range of neurological symptoms, such as anxiety, ataxia, and headaches, in addition to body image dissatisfaction, may occur in patients with CD, especially adolescents (Daldaban Sarıca et al.). It is critical to recognize mood disorder and body image dissatisfaction symptoms early in order to improve the general well-being of adolescents with CD and provide appropriate patient management. Periodic follow-up is necessary to identify mood-related symptoms and assess the teenagers' body acceptance.

The wide spectrum of clinical manifestations makes CD challenging to identify, often leading to significant delays in diagnosis. According to reports, the delay in diagnosing CD may range from months to over a decade (1, 7, Naredi Scherman et al.). It is crucial to diagnose CD early in order to avoid long-term consequences. The sole curative option is a lifelong gluten-free diet (GFD).

Despite good adherence to a GFD, a recent study showed nutritional inadequacies in children with CD (Ekşi et al.). A nutritional assessment should be performed at each visit. Any identified deficiencies, such as those involving vitamin D, folate, B vitamins, iron, calcium, or zinc should be addressed.

Accidental gluten ingestion is a significant issue troubling patients and physicians alike. It has been reported that one-third of children with CD on a GFD suffer from acute gastrointestinal symptoms such as vomiting and nausea as a result of accidental gluten ingestion (Pjetraj et al.). To enhance quality of life by preventing accidental exposure to gluten in individuals with CD, it is critical to educate, monitor and regulate accidental gluten consumption.

We hope that you will enjoy reading this special issue with its new studies on pediatric CD. We believe that these articles, which were evaluated through a thorough peer review and deemed appropriate for publication by the editors, contribute significantly to the existing literature on celiac disease.
